# Phagocytosis of *Streptococcus pyogenes* by All-*Trans* Retinoic Acid-Differentiated HL-60 Cells: Roles of Azurophilic Granules and NADPH Oxidase

**DOI:** 10.1371/journal.pone.0007363

**Published:** 2009-10-06

**Authors:** Pontus Nordenfelt, Susanne Bauer, Per Lönnbro, Hans Tapper

**Affiliations:** Section for Clinical and Experimental Infection Medicine, Department of Clinical Sciences, Lund University, Lund, Sweden; University of Birmingham, United Kingdom

## Abstract

**Background:**

New experimental approaches to the study of the neutrophil phagosome and bacterial killing prompted a reassessment of the usefulness of all-*trans* retinoic acid (ATRA)-differentiated HL-60 cells as a neutrophil model. HL-60 cells are special in that they possess azurophilic granules while lacking the specific granules with their associated oxidase components. The resulting inability to mount an effective intracellular respiratory burst makes these cells more dependent on other mechanisms when killing internalized bacteria.

**Methodology/Principal Findings:**

In this work phagocytosis and phagosome-related responses of ATRA-differentiated HL-60 cells were compared to those earlier described in human neutrophils. We show that intracellular survival of wild-type *S. pyogenes* bacteria in HL-60 cells is accompanied by inhibition of azurophilic granule–phagosome fusion. A mutant *S. pyogenes* bacterium, deficient in M-protein expression, is, on the other hand, rapidly killed in phagosomes that avidly fuse with azurophilic granules.

**Conclusions/Significance:**

The current data extend our previous findings by showing that a system lacking in oxidase involvement also indicates a link between inhibition of azurophilic granule fusion and the intraphagosomal fate of *S. pyogenes* bacteria. We propose that differentiated HL-60 cells can be a useful tool to study certain aspects of neutrophil phagosome maturation, such as azurophilic granule fusion.

## Introduction

The human promyelocytic HL-60 cell line continuously proliferates in suspension culture and can by various agents be induced to differentiate into granulocytes, monocytes, macrophages or eosinophils [1]. The original cells were isolated and described in 1977 by Collins et al. [2] and is reported to have Fc receptors (20, 000 per cell) with high affinity towards human IgG1 and IgG3 (5-10 nM) ([3]). The proportion of Fc receptor-positive cells also increase (from ∼20% to ∼50%) during differentiation with retinoic acid [4]. In this paper, we used all-*trans* retinoic acid (ATRA) to induce a neutrophil-differentiated phenotype that contains azurophilic granules but lacks the specific granules [5] and other granule types that are formed late in the granulocytic maturation process [6], [7]. To study the mechanisms regulating the fusion of azurophilic granules with phagosomes, important for the efficient killing of bacteria by human neutrophils, we thus reasoned that it would be advantageous to use neutrophil-differentiated HL-60 cells. For such studies, we first needed to show that neutrophil-differentiated HL-60 cells can efficiently phagocytose bacteria and that the latter can be killed inside phagosomes that fuse with azurophilic granules.

The azurophilic granules contain bactericidal substances such as various proteases, defensins and anti-microbial peptides [8] that can be delivered to phagosomes by fusion. Neutrophils, but not HL-60 cells, also have additional granule types that can fuse with phagosomes, adding further to the phagosome antibacterial arsenal by, e.g., enabling activation of an intraphagosomal production of oxidants [9]. The neutrophil respiratory burst requires several components to function. One is the membrane-bound flavocytochrome *b*_558_, which is composed of a large glycoprotein (gp91^phox^) and a smaller protein (p22^phox^) [10]. The catalytic core of the oxidase is the gp91^phox^ subunit, also called Nox2, after NADPH oxidase [11], [12]. The other components involved can be found in the cytosol and consist of p40^phox^, p47^phox^, p67^phox^ and the small GTPases Rac1 and Rac2 [13], [14]. For a NADPH oxidase review, see Vignais [15]. Because HL-60 cells are incomplete in their granule arsenal, their use might be informative when investigating the involvement of the plasma membrane-localized respiratory burst [16], [17] in the killing of bacteria. In neutrophils, the flavocytochrome *b*_558_, i.e. the membrane-bound component of the NADPH oxidase, is not only localized to the membranes of the specific granules [18] (approximately 85%), but also to secretory vesicles and the plasma membrane [19]. HL-60 cells contain neither specific granules nor secretory vesicles. Despite the lack of granular flavocytochrome *b*_558_, some phagosomal oxidase activity can still occur in HL-60 cells during phagosome formation or, later, by the delivery of plasma membrane-derived oxidase components to the phagosome by fusion processes. Therefore, the question whether or not the respiratory burst is important for the killing of *S. pyogenes* was also addressed in this study.

*Streptococcus pyogenes* is a Gram-positive human pathogen that causes a wide range of diseases, from uncomplicated pharyngitis and pyoderma to severe and life-threatening invasive diseases such as sepsis and streptococcal toxic shock syndrome [20]. *S. pyogenes* bacteria use multiple strategies to avoid being killed by host cells [21] and have been known for many years to be able to survive incubation in human non-immune blood. This ability has been ascribed, at least partly, to an antiphagocytic effect of M and M-like proteins expressed on the bacterial surface [22]. However, we have shown that *S. pyogenes* bacteria are phagocytosed efficiently by human neutrophils and that avoidance of killing is accompanied by an inhibited fusion of azurophilic granules with the phagosome [23], [24]. In contrast, the isogenic mutant strain, BMJ71, lacking the *mga* regulon which codes for the virulence factors M protein, protein H, SIC and C5a peptidase [25], is rapidly killed and degraded inside phagosomes that avidly fuse with azurophilic granules.

In the following, we demonstrate that valuable information can be obtained by using ATRA-differentiated HL-60 cells in studies of neutrophil phagocytosis.

## Results

### All-*trans* retinoic acid enhances the phagocytic ability of HL-60 cells

**Figure 1:**
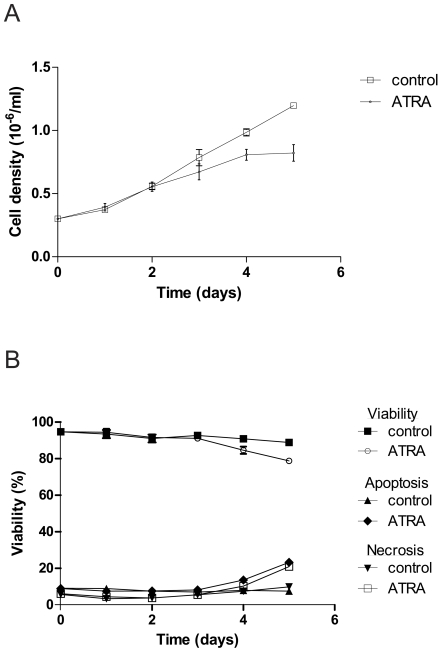
Effects of ATRA-induced differentiation of HL-60 cells. A. Cell growth during differentiation. HL-60 cells were treated with ATRA during five days and cell growth was measured by using a Bürker chamber. Error bars show SEM, based on four cell counts. B. Viability and apoptosis/necrosis during differentiation. Viability was measured by evaluation of trypan blue exclusion using a Bürker chamber. Error bars (smaller than symbols) show SEM, based on four cell counts. To measure apoptosis and necrosis, samples were stained with Alexa 488-conjugated annexin V and propidium iodide. Data shown were obtained using flow cytometry (30,000 cells per condition). Similar data were obtained by quantitative fluorescence microscopy (100 cells per condition) where the criteria for being classified as an apoptotic cell were distinct plasma membrane staining of annexin V, and no propidium iodide staining. Cells positive for both markers were classified as necrotic.

**Figure 2:**
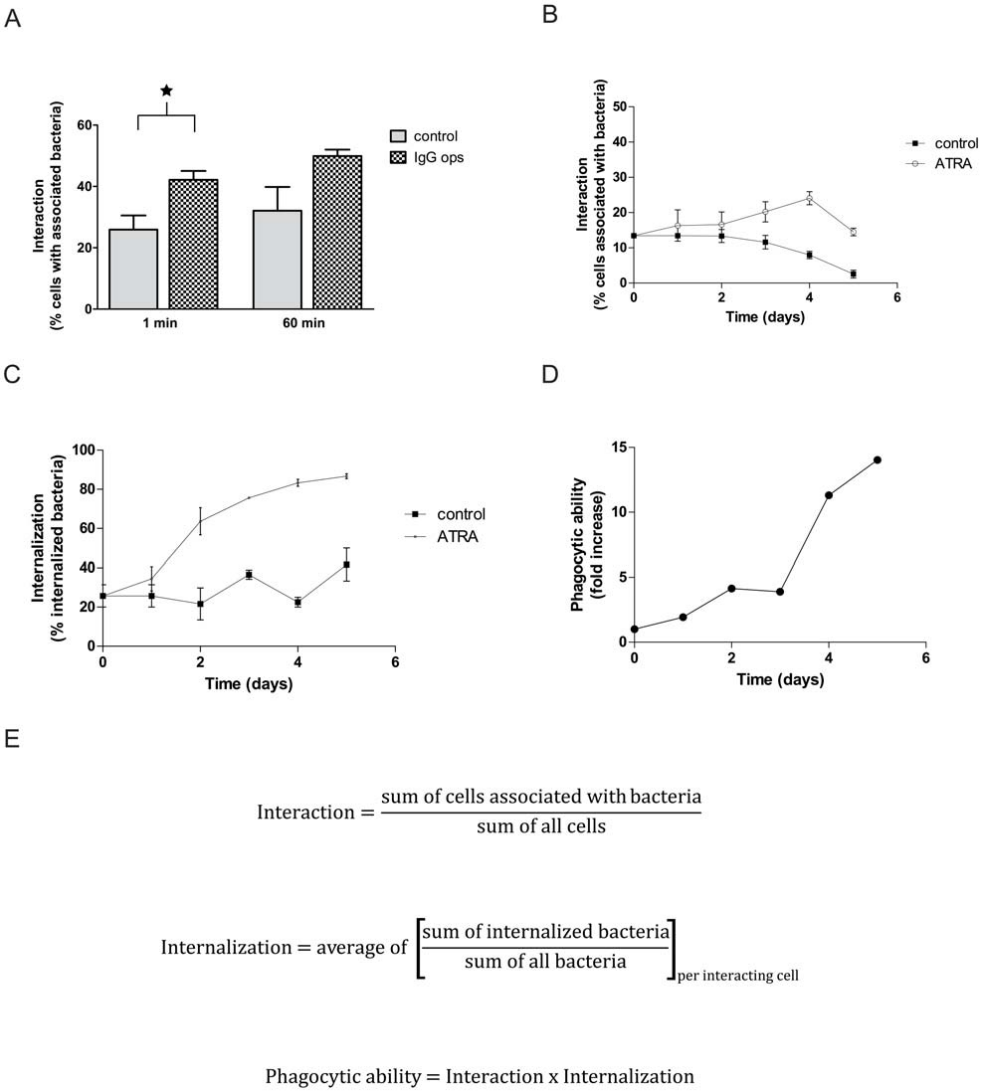
Phagocytic ability is induced by ATRA differentiation. A. Effect of opsonization on interaction. Differentiated HL-60 cells were allowed to interact at 37°C with Oregon Green-labeled BMJ71 bacteria, either IgG-opsonized (1 mg/ml) or not, at a bacteria/cell ratio of 10∶1. After a synchronized presentation, the samples were incubated at 37°C as indicated. Analysis was by flow cytometry. An interaction ratio was calculated by dividing the number of cells interacting with Oregon Green-labeled bacteria with the total number of cells. Error bars show SEM, based on a total of three experiments; *  = p<0.05. B–C. Interaction and internalization during differentiation. The ability of HL-60 cells to associate with and to internalize BMJ71 bacteria was measured. Oregon Green-labeled and IgG-opsonized bacteria were allowed to interact for 5 min with the cells at a bacteria/cell ratio of 2∶1. Analysis was by fluorescence microscopy. Error bars represent SEM of two separate experiments. D. Phagocytic ability induced by ATRA-treatment. Fold increase of phagocytic ability arrived at by normalizing the phagocytic ability of ATRA-treated cells to the corresponding control sample. E. Basis for calculations. The different formulas used for the analysis of data in the figures B–D are presented. Interaction is defined as the fraction of all cells that are associated with at least one bacterium. Interacting cells were further analyzed to determine how large a fraction of the associated bacteria was intracellular. The term phagocytic ability was constructed to take interaction as well as internalization into account.

We investigated the effects of ATRA treatment on HL-60 cells over a five-day period. Cell density was affected by ATRA treatment, and a reduced growth rate (in accordance with induction of differentiation) compared to control cells was observed (Fig. 1A). Also viability and apoptosis was monitored, showing a slightly reduced viability for ATRA-treated cells on day 4 and 5 with a concurrent increase in apoptosis and necrosis (Fig. 1B). Having ensured that the differentiated cells were in good health, we proceeded to investigate the phagocytic ability of these cells. Mutant (BMJ71) *S. pyogenes* bacteria were presented to HL-60 cells at 37°C by a synchronized presentation protocol. In order to study a general and defined uptake mechanism we chose to focus on Fc-receptor mediated phagocytosis of IgG-opsonized prey, since these are present in HL-60 cells [3]. The effect of opsonization on the interaction of cells with bacteria is shown in Figure 2A. Similar to neutrophils, a larger proportion of the HL-60 cells interacted with bacteria that had been opsonized with human IgG. This enhanced interaction was observed also using the wild-type strain AP1 (not shown). An interaction ratio was calculated by relating the number of cells with attached and/or phagocytosed Oregon Green-labeled bacteria to the total number of cells. The interaction of HL-60 cells with bacteria was slightly less efficient than was the case for human neutrophils under comparable assay conditions in a previous report [24].

Two parameters were studied: the interaction of cells with bacteria and the internalization of cell-associated prey. Figure 2B shows that the interaction is slightly increased during differentiation with ATRA. In contrast, the control cells decrease their interaction ability. Looking at internalization, there is an increase following ATRA treatment as compared to control cells (Figure 2C). To better assess the phagocytic ability, we combined interaction and internalization data. This was normalized to that of control cells, illustrating the ATRA-induced phagocytic ability over time (Fig. 2D). Figure 2E presents the basis for calculations used in figures 2B, 2C and 2D.

### Intracellular survival of *S. pyogenes* bacteria and inhibition of azurophilic granule–phagosome fusion

**Figure 3:**
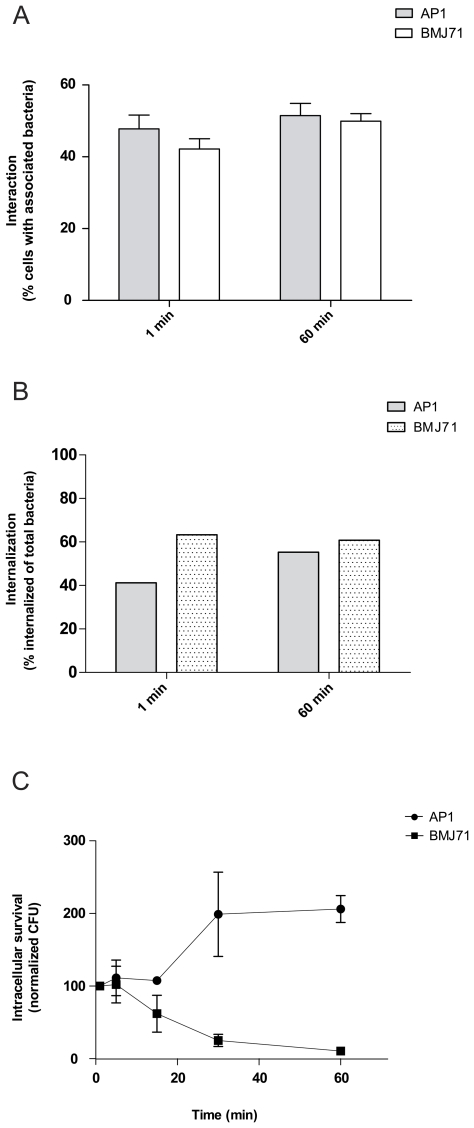
Intracellular survival of *S. pyogenes* bacteria. A. Interaction. Differentiated HL-60 cells were allowed to interact at 37°C with Oregon Green-labeled IgG-opsonized (1 mg/ml) AP1 and BMJ71, at a bacteria/cell ratio of 10∶1. After a synchronized presentation, the samples were incubated at 37°C as indicated. Analysis was by flow cytometry. An interaction ratio was calculated by dividing the number of cells interacting with Oregon Green-labeled bacteria with the total number of cells. Error bars show SEM, based on a total of three experiments. B. Internalization. After phagocytosis as in A, the internalization of bacteria was determined by immunofluorescence microscopy using non-permeabilized and permeabilized conditions and anti-*S. pyogenes* antibodies. For each condition, at least 100 cells were counted. A representative experiment is shown. C. Intracellular survival. Differentiated HL-60 cells were allowed to phagocytose AP1 and BMJ71 bacteria at 37°C, bacteria/cell ratio 10∶1. After a synchronized presentation, the samples were incubated at 37°C as indicated, before killing of extracellular bacteria by PlyC. Intracellular survival of bacteria was determined by diluting HL-60 lysates and counting the number of colonies formed after overnight growth at 37°C. Data shown are expressed as the CFU ability relative to the values at 1 min. Error bars show SEM, based on a total of three experiments.

We have previously described that wild-type *S. pyogenes* (AP1 strain) can survive phagocytosis by neutrophils [24] and that this can be correlated with an inhibition of azurophilic granule–phagosome fusion [23]. Here, we similarly show that AP1 bacteria survived better inside differentiated HL-60 cells than did mutant BMJ71 bacteria, see Figure 3C. Using fluorescence microscopy to distinguish between phagocytosed and surface-associated bacteria we wanted to see whether this could be explained by a difference in internalization rate. The wild-type AP1 strain was found to be slightly more resistant to internalization at early time points (Fig. 3B), but after one hour no difference could be seen between the two bacterial strains. The proportion of bacteria interacting with HL-60 cells was also compared (Fig. 3B) and no significant difference could be noted between AP1 bacteria and BMJ71 bacteria. A more efficient interaction or internalization of BMJ71 bacteria thus cannot explain the survival benefit of AP1 bacteria in phagosomes.

**Figure 4:**
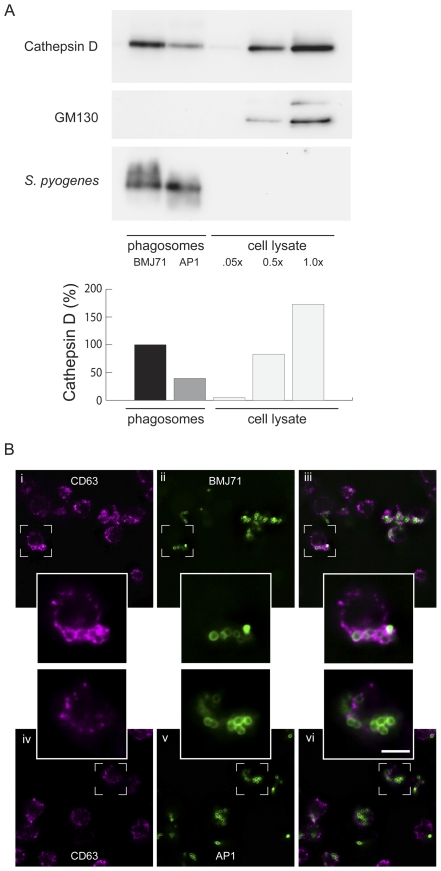
Fusion of azurophilic granules with phagosomes containing *S. pyogenes* bacteria. A. Western blot of isolated phagosomes and cell lysate. Differentiated HL-60 cells were allowed to phagocytose IgG-opsonized, heat-killed and magnetically labeled *S. pyogenes* bacteria for 20 min at a bacteria/cell ratio of 5∶1. Following washes, the cells were lysed by nitrogen cavitation and phagosomes were retrieved by magnetic selection. Equal amounts of phagosomes were loaded and probed with antibodies against cathepsin D (azurophilic granule content marker) and GM130 (Golgi marker) with anti-*S. pyogenes* as loading control. Increasing amounts of cell lysate (relative protein content.05×, 0.5× and 1.0×) was used as a control. B. Azurophilic granule–phagosome fusion. Differentiated HL-60 cells were allowed to phagocytose opsonized Oregon Green-labeled *S. pyogenes* bacteria, either the BMJ71 (i-iii) or the AP1 (iv–v) strains, at a bacteria/cell ratio of 10∶1. After a synchronized presentation, the samples were incubated at 37°C for 5 min, fixed and incubated with antibodies directed against CD63, subsequently detected using Alexa 594 F(ab')_2_ fragments. Single deconvolved focal planes from serial z-stacks were taken from the mid-part of the HL-60 cells. Oregon Green staining shows the localization of bacteria (ii, v). Magenta staining shows the localization of the azurophilic granule membrane marker CD63 (i, iv). The images are also presented as merged (iii, vi). Size bar: 5 µm.

Next, the fusion of azurophilic granules with bacteria-containing phagosomes was investigated. We employed a technique based on magnetic selection to isolate bacteria-containing phagosomes [26]. In Figure 4A, a reduced delivery of azurophilic content to phagosomes containing AP1 bacteria is demonstrated by Western blot. The membrane was probed with antibodies against cathepsin D (azurophilic granule content marker) and GM130 (Golgi marker, used as a negative control). As shown in the figure, the cathepsin D band of BMJ71 phagosomes is stronger than that of AP1 phagosomes. GM130 is only visible in the cell lysate lanes. Protein content was determined by EZQ (Invitrogen), equal loading being verified by post-blot UV-scanning. As an additional loading control, the membrane was probed with an antibody recognizing *S. pyogenes*, revealing similar amounts of bacterial protein in the phagosome lanes. We also employed immunofluorescence microscopy, but since it is difficult to quantitate fusion to small phagosomes in whole cells we only used this method qualitatively. To evaluate the experiments, cells were inspected for bacteria surrounded by fluorescent ring-like structures that indicated fusion of azurophilic granules with phagosomes. For the BMJ71 strain, such fusion patterns were observed in a majority of the cells. For the AP1 strain, a smaller fraction of the cells displayed a pattern consistent with azurophilic granule–phagosome fusion (Fig. 4C).

Taken together, the data in Figure 4 suggests that wild-type *S. pyogenes* bacteria can reduce the fusion propensity of phagosomes in HL-60 cells.

### Minor contribution of the plasma membrane respiratory burst to killing of *S. pyogenes* bacteria

**Figure 5:**
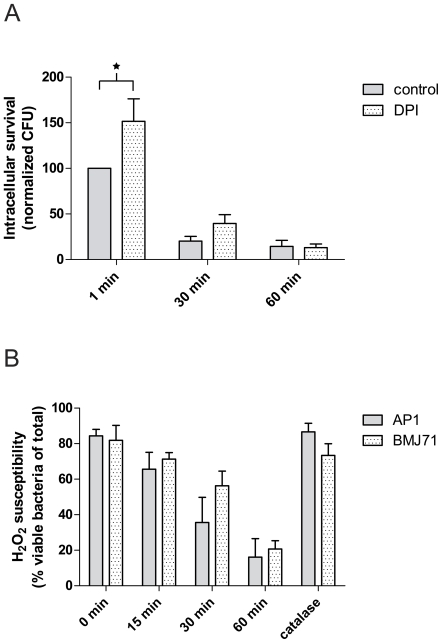
Killing of BMJ71 bacteria does not require Nox2 activation. A. Intracellular killing with inhibited oxidase. Differentiated HL-60 cells were allowed to phagocytose BMJ71 bacteria at 37°C, bacteria/cell ratio 10∶1, in the presence or absence of 10 µM DPI. After a synchronized presentation, the samples were incubated at 37°C as indicated, before killing of extracellular bacteria by PlyC. Intracellular survival of bacteria was determined by diluting HL-60 lysates and counting the number of colonies formed after overnight growth at 37°C. Data shown are expressed as the CFU ability relative to the control value at 1 min. Error bars show SEM, based on a total of five experiments. A significant difference was found between control and DPI-treated cells at the 1 min time point, p<0.05. B. H_2_O_2_ susceptibility of *S. pyogenes*. BMJ71 and AP1 bacteria were incubated with 1.5% H_2_O_2_ at 37°C as indicated. The samples were stained using a bacterial viability kit (Viagram) and analyzed by fluorescence microscopy. As a control, catalase (1,000 U/ml) was added. At least 100 bacteria per condition were analyzed. Error bars show SEM, based on a total of three experiments.

In neutrophils, high concentrations of superoxide and hydrogen peroxide is generated in the phagosome [27]. Earlier, we reported that similar amounts of oxidative metabolites were observed to be generated during neutrophil phagocytosis of the AP1 and BMJ71 strains [23]. To investigate the role of respiratory burst metabolites for the killing of bacteria, we performed survival assays using differentiated HL-60 cells treated with the oxidase-inhibitor DPI [28]. Early control experiments showed no effect on intracellular survival of AP1 bacteria so we used instead the less virulent BMJ71 strain. Such treatment did only slightly increase the survival of the bacteria, see Figure 5A. Looking at later time points, it would appear that activation of the respiratory burst is not the only mechanism responsible for the killing of BMJ71 bacteria in HL-60 cells. However, Nox2 activation at the plasma membrane might contribute to the rapid killing of BMJ71 bacteria during phagocytosis by differentiated HL-60 cells. Therefore, we investigated the susceptibility of *S. pyogenes* bacteria to the respiratory burst product H_2_O_2_. The bacteria were killed by H_2_O_2_ treatment (Figure 5B). Importantly, the wild-type AP1 strain was not more resistant than the BMJ71 strain to killing by H_2_O_2_.

**Figure 6:**
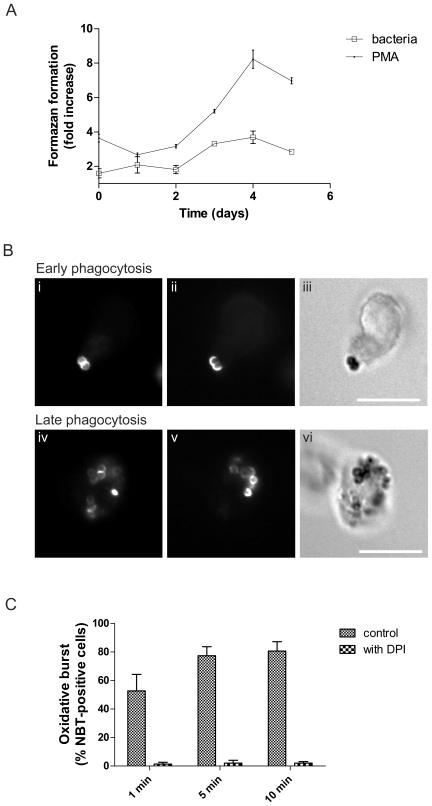
ATRA-induced oxidative ability. A. Induction of Nox2 activation during differentiation. Cells were allowed to phagocytose IgG-opsonized BMJ71 bacteria for 5 min (bacteria/cells, 2∶1), or were stimulated with 160 nM PMA, in the presence of 1 mg/ml NBT. Measurement of absorbance was used to quantitate the intracellular respiratory burst. Data are presented as the ratio between formazan formation in ATRA-treated cells compared to control cells. B. Localization of formazan formation. Differentiated HL-60 cells interacting with IgG-opsonized Oregon Green-labeled BMJ71 bacteria were incubated with 1 mg/ml NBT. A bacteria/cell ratio of 10∶1 was used. Respiratory burst activity was indicated by blue-black formazan precipitates, visible by light microscopy (iii, vi). Oregon Green fluorescence (i, iv) shows the total number of bacteria, and anti-streptococcal staining (ii, v) shows the location of extracellular bacteria. In the upper panel, precipitate formation on cell-adherent bacteria is illustrated. The lower panel shows that NBT precipitates may also be found on internalized bacteria. Size bar: 10 µm. C. Quantitation of respiratory burst-positive cells. The diagram shows the proportion of bacteria-interacting cells that display formazan precipitates and the effect of DPI (10 µM). At least 50 cells per sample were analyzed. Error bars show SEM, based on a total of three experiments.

Having observed only a minor effect of the DPI treatment on the intracellular survival of *S. pyogenes* bacteria, we wanted to confirm the efficient inhibition of respiratory burst activity in differentiated HL-60 cells by DPI. We therefore investigated stimuli-triggered formazan formation during differentiation. This response proved to increase over the studied period, with a peak on day 4 (Fig. 6A). Fluorescence microscopy was employed in order to locate regions of respiratory burst activity during phagocytosis in HL-60 cells. As can be seen in figure 6B, black formazan precipitates, indicating oxidation of NBT by superoxide anion, could be observed associated with both extra- and intracellular bacteria, although precipitates were more pronounced on the former. Precipitates could be detected already after one minute. At later times, most of the cells interacting with bacteria displayed evidence of respiratory burst activity. Precipitate formation was prevented in samples incubated with DPI (Fig. 6C). A triggering of a DPI-inhibitable respiratory burst in HL-60 cells by BMJ71 bacteria was also observed using flow cytometric analysis of cells pre-loaded with dihydrorhodamine 123 ([29], [30] data not shown). Taken together, despite a localized phagocytosis-induced respiratory burst in HL-60 cells, this response appears to play a minor role in the killing of *S. pyogenes* bacteria.

## Discussion

We have examined the interaction of *S. pyogenes* bacteria with differentiated HL-60 cells. The goal was to better understand neutrophil phagocytosis and the mechanisms by which wild-type AP1 bacteria interfere with intracellular killing. We suspected the involvement of azurophilic granules, with their arsenal of proteases and other antimicrobial substances, as well as the production of respiratory burst metabolites triggered by NADPH oxidase activation.

Initial experiments were designed to characterize our experimental system. The agents most commonly used for the differentiation of HL-60 cells towards a neutrophil-like state are DMSO and ATRA [31], but others have also been used [32], [33]. During differentiation, various characteristics of the HL-60 cell phenotype change, e.g. the appearance of neutrophil cell surface antigens such as CD11b [34] and CD66 [35], [36], as well as the downregulation of CD71 [37]. We opted to use ATRA, which tends to yield a high percentage of neutrophil-like cells [38]. Using low-passage cells (less than two months from stock) was also a conscious decision, since extended continuous cell culture may affect the response to differentiation agents [38]. Usually seen as a lack of growth arrest, this was not observed in our experiments, thus indicating “true” HL-60 cells.

There are several ways to evaluate phagocytic function. The most commonly used parameter is called the phagocytic index and was introduced by Silverstein and co-workers [39], and is also known as the phagocytosis product [40]. It consists of two factors; the percentage of cells that are associated with prey (adhered or internalized) and the average number of prey per cell. This works well in most cases when studying professional phagocytes, but it does not give any information about the ability to internalize *per se*. In this work we are looking at the potential gain of phagocytic ability during differentiation. In our view, this could be an increase of association with prey (or interaction, as we call it), an increase of internalization of prey or both. Our results show that both of these parameters are affected, and we therefore constructed a composite of these which we denote phagocytic ability. The conclusion that we draw from differentiation with ATRA is that the treatment enhanced the phagocytic ability of the cells to a high degree.

**Figure 7:**
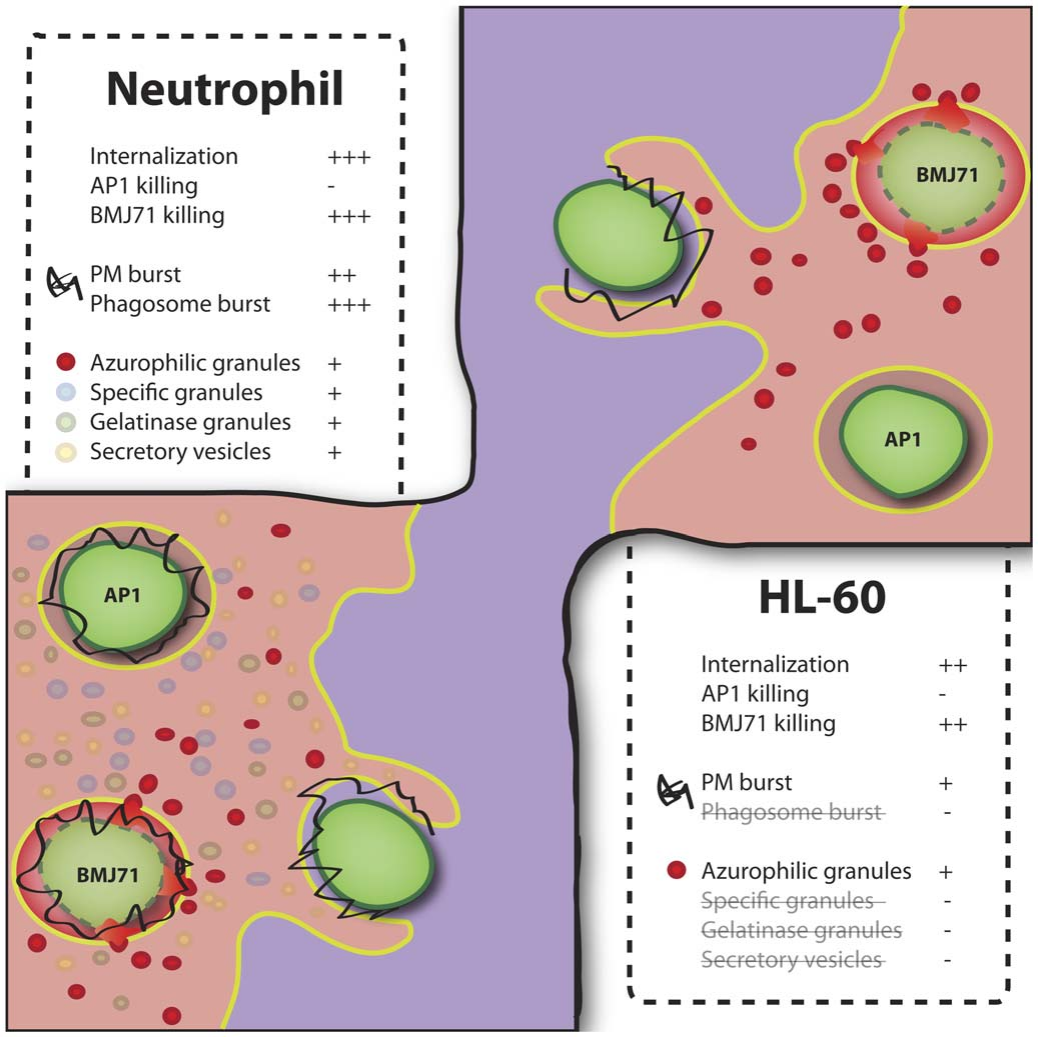
ATRA-differentiated HL-60 cells as a model for neutrophil phagocytosis. Both neutrophils and differentiated HL-60 cells are able to internalize and kill IgG-opsonized prey within minutes. However, phagosome maturation differs for the two cell types because HL-60 cells have only azurophilic granules (depicted in red) and lack other granule types. Also, Nox2 activity (depicted in black) is lower in HL-60 cells and located at the plasma membrane. These deficiencies of ATRA-differentiated HL-60 cell lines can be exploited for the study of the roles of azurophilic granules and Nox2 during phagocytosis. In the figure, the different fates of two strains of *S. pyogenes* bacteria in both neutrophils and HL-60 cells are shown. In both cell types, the killing of the BMJ71 strain is accompanied by the avid fusion of azurophilic granules, whereas less fusion and less killing is observed for the AP1 strain.

The use of a cell line for phagosome studies provides several benefits, both practical and scientific: it reduces experimental variation due to heterogeneity [41]; it is possible to transfect HL-60 cells; large number of cells can easily be obtained; and it represents a reductionist system where several of the neutrophil granule types are absent (phagosomal oxidase assembly is defective because of the lack of specific granules). Further, for phagosome isolation experiments, the use of HL-60 cells offers several additional advantages, such as reduction of total experiment time and increased reproducibility. Important phagosome maturation differences between the HL-60 and neutrophil models are summarized in Figure 7.

In accordance with recently published data on the intracellular survival of *S. pyogenes* bacteria in neutrophils [23], an inhibition of the fusion of azurophilic granules with phagosomes containing wild-type AP1 bacteria was observed in HL-60 cells. In contrast, massive fusion occurred on phagosomes containing the mutated strain BMJ71, lacking M and M-like proteins. Phagosome isolation allowed a quantitative analysis of azurophilic granule–phagosome fusion. A potential drawback is that we used heat-killed bacteria for this. However, we have earlier reported that effects on intracellular trafficking persist even after heat treatment of bacteria, albeit to a lesser degree [23], [42]. The reduced amount of azurophilic granule-marker found on phagosomes containing wild-type bacteria as compared to those containing the mutant provided further evidence to the hypothesis that wild-type *S.pyogenes* bacteria interfere with phagosome maturation.

In neutrophils, respiratory burst products such as O_2_^−^ and H_2_O_2_ can be formed in phagosomes [43], and this is the case with both BMJ71- and AP1-containing phagosomes [23]. AP1-containing phagosomes do not readily fuse with azurophilic granules and lack of fusion leads to a lack of the azurophilic granule component MPO, which catalyzes reactions between H_2_O_2_ and, e.g., halides into more toxic oxidative products such as hypochlorite [44]. The phagosomes containing BMJ71 bacteria would therefore generate larger amounts of more potent oxidative metabolites. This could be one factor contributing to the observed difference in survival between the two bacterial strains.

Because specific granules are absent in HL-60 cells, the phagosomal respiratory burst activity in these cells ought to be lower than in neutrophils since the deposition of specific granule NADPH oxidase components on the phagosome is not possible. Instead, during differentiation, HL-60 cells develop an ability to mount a plasma membrane oxidative burst in the vicinity of forming phagosomes. After showing that the bacteria used were sensitive to oxygen metabolites, the effect of the oxidase inhibitor DPI on intracellular survival of bacteria was assessed. The survival was only affected to a small extent and only at the early stage of phagocytosis.

In conclusion, the survival of *S. pyogenes* bacteria in neutrophil-differentiated HL-60 cells seems to be caused by an inhibition of azurophilic granule fusion with phagosomes and not primarily by inhibition of the respiratory burst. The intracellular fate of wild-type and mutant *S. pyogenes* strains in neutrophils is very similar to what is observed in HL-60 cells, and it is therefore tempting to suggest that the relevant antibacterial mechanisms in the HL-60 cell line might be similar to those used by neutrophils. In other words, a major role for azurophilic granules and a limited role for plasma membrane respiratory burst activity should apply for both cell types. Further studies are needed to clarify the role of the neutrophil NADPH oxidase as a means of defense against *S. pyogenes* intracellular persistence. Finally, we have demonstrated that the HL-60 cell, when used in parallel with human neutrophils, provides a useful model for phagosome maturation studies, in which the respiratory burst (and granule types other than the azurophilic) does not significantly contribute to bacterial killing.

## Materials and Methods

### Bacteria

The wild-type *S. pyogenes* AP1 (40/58) strain of the M1 serotype was provided by the World Health Organization Streptococcal Reference Laboratory in Prague, Czech Republic. The *mga*-regulon deficient *S. pyogenes* strain BMJ71, was generated from the wild-type serotype as previously described [25]. The bacteria were grown as described by Staali et al [24] and opsonized with pooled human IgG (Sigma-Aldrich, Stockholm, Sweden). For some experiments, bacteria were stained with 5 µM Oregon Green 488-X succinimidyl ester (Invitrogen, Copenhagen, Denmark) for 30 min in the dark at room temperature. Excess fluorochrome was then removed by washing the bacteria three times in PBS. The stained bacteria were kept on ice until use.

### HL-60 cells

HL-60 cells were acquired from the ATCC and were kept in low passage (<2 months) and then exchanged for freshly thawed aliquots. In accordance with the protocol of Breitman et al. [6], seeding of HL-60 cells was performed in l-glutamine-containing RPMI 1640 medium (PAA Labs, Gothenburg, Sweden), supplemented with 10% fetal bovine serum (Gibco, Copenhagen, Denmark) and 1 µM ATRA (Sigma-Aldrich, Stockholm, Sweden). The cell concentration was 0.3–0.4·10^6^/ml and the viability was over 95%. The cells were kept in 5% CO_2_ atmosphere at 37°C for four days and were then harvested. No antibiotics were used. The viability of the differentiated cells was 75–80%, as determined by trypan blue exclusion. After harvesting (20·10^6^/ml), the HL-60 cells were put on a rotator (8 rpm) at room temperature. All experiments were performed in Na-medium (5.6 mM glucose, 127 mM NaCl, 10.8 mM KCl, 2.4 mM KH_2_PO_4_, 1.6 mM MgSO_4_, 10 mM HEPES, 1.8 mM CaCl_2_; pH adjusted to 7.3 with NaOH), within 30 min of harvesting.

### Phagocytosis

Phagocytosis of bacteria was performed in Na-medium which was preheated to 37°C. Different bacteria/cell ratios were used for different assays, varying between 2∶1–10∶1 (see figure legends). These were chosen empirically to compensate for differences in presentation techniques employed. To synchronize interaction, bacteria and cells were pelleted in a microcentrifuge (12,000 *g*, 30 s), followed by an additional 30 s incubation at 37°C. The pellet was then resuspended and the cells were further incubated at 37°C. Phagosome maturation was halted by putting the samples on ice. To ensure equal treatment of the samples, an 8-channel multi-pipet was used for resuspension. In some experiments (figures 2B, 2C, 4B and 6A) the presentation step was repeated to increase interaction efficiency.

### Intracellular survival assays

To study intracellular survival of *S. pyogenes*, the bacterial strains were first sonicated (XB2 sonicator bath, Grant) to reduce bacterial aggregation. Before presentation to cells, they were IgG-opsonized (1 mg/ml) and then gently centrifuged (200 *g*, 2 min, swing-out) to remove any remaining aggregates (checked by microscopy). After equilibration at 37°C, differentiated HL-60 cells were presented to the bacteria by centrifugation (12,000 *g*, 30 s, fixed angle). The phagocytic process was halted by placing the samples on ice and extracellular bacteria were killed by incubating the samples with PlyC, a streptococcal C1 bacteriophage lysin (functionally a murein hydrolase [45], [46]), at 1.5 U/µl for 15 min on ice, followed by thorough washing. The HL-60 cells were lysed by incubating the samples for 20 min with 2% saponin. The samples were diluted in distilled water and plated on Todd-Hewitt agar plates. Following an overnight incubation at 37°C, the number of colony forming units (CFU) was determined.

### Phagosome isolation

The cells were allowed to interact with the bacteria at a bacteria/cell ratio of 5∶1 and after 20 min of phagocytosis the maturation was halted by placing the samples on ice. Bacteria-containing phagosomes were prepared using a method of magnetic selection as described by Lönnbro et al. [26].

### Western blot

SDS-PAGE was performed using a modified protocol of Laemmli [47] made according to instructions for NuPAGE gels (4–12% Bis-Tris, Invitrogen) and PVDF membranes (Millipore). The membranes were probed using antibodies against cathepsin D (Santa Cruz Technology, Santa Cruz, CA), GM130 (Affinity Bioreagents, CO, USA) and group A streptococci (Biogenesis, Poole, England). Western blots were developed with Super Signal West Dura Extended (Pierce). PageRuler Plus (Fermentas) was used as molecular weight standard.

### Flow cytometry

Flow cytometric analysis was performed using a FACSCalibur flow cytometer (Becton-Dickinson) equipped with a 15 mW argon laser tuned at 488 nm. For each sample at least 20,000 events were recorded and the results were analyzed using the CellQuest Pro software (Becton Dickinson) and FlowJo 8.8.6 (Tree Star).

### Immunofluorescence microscopy

In figure 4, samples were treated and analyzed as described by Staali et al [24]. A monoclonal antibody against CD63 (1∶800, (Santa Cruz Technology, Santa Cruz, CA)) and a polyclonal goat antibody against *S. pyogenes* (1∶1000, (AbD Serotec, Düsseldorf, Germany) were used as primary antibodies. As secondary antibodies an Alexa Fluor 594 anti-mouse antibody or an Alexa Fluor 488 anti-goat antibody (1∶600, (Invitrogen, Copenhagen, Denmark)) were used. Acquisition of images were performed using a fluorescence microscope (Nikon Eclipse TE300 equipped with a Hamamatsu C4742-95 cooled CCD camera, using a Plan Apochromat 100X objective with NA 1.4). To increase resolution, z-series were captured and out-of-focus light was removed by deconvolution (NIS-Elements 3, Nikon). This was performed with a calibrated point spread function to limit errors introduced by the microscope setup.

To analyze interaction and internalization during phagocytosis (cf. Fig. 2B and 2C) Oregon Green-labeled bacteria were used. The opsonizing IgG on non-internalized bacteria were visualized by the addition of anti-human Cy3-conjugated F(ab')_2_ fragments (Jackson Immunoresearch, Baltimore, PA).

### NBT assays

Oregon Green-labeled bacteria were suspended in PBS containing 1 mg/ml NBT (Roche Diagnostics, Bromma, Sweden). After allowing phagocytosis as described above, the samples were fixed using 2% PFA. To distinguish extracellular bacteria from intracellular, the former were stained using a goat anti-streptococcal antibody (1∶600) and Alexa Fluor 594 F(ab')_2_ fragment of anti-goat IgG (1∶600). The presence of blue-black formazan precipitates, caused by the reduction of NBT by the superoxide anion (O_2_^−^) [48], was quantified by microscopy. In order to quantitate the intracellular respiratory burst, cells were allowed to phagocytose IgG-opsonized BMJ71 bacteria for 5 min or were treated with 160 nM phorbol 12-myristate 13-acetate (PMA, Sigma-Aldrich, Stockholm, Sweden) [49] in the presence of 1 mg/ml NBT. The samples were treated with 70% methanol followed by 2 M KOH, after which the formazan precipitates were dissolved using concentrated DMSO as described by Mollinedo et al. Finally, absorbance (600 nm and 450 nm) was measured using a Victor3 plate reader (PerkinElmer).

### Oxidant susceptibility assay

H_2_O_2_ was added to 2·10^9^ bacteria at a final concentration of 1.5% (v/v). Samples were incubated at 37°C for 15, 30 and 60 minutes, respectively, after which catalase (1000 U/ml) was added to stop the reaction as described by Liu et al. [50]. Samples were fixed using 1% PFA and stained using the Viagram Viability Kit (Invitrogen, Copenhagen, Denmark). Bacterial viability was analyzed by fluorescence microscopy. Alternatively, analysis of H_2_O_2_-treated bacteria (non-fixed) was performed by counting colonies after 16 h incubation on Todd-Hewitt agar at 37°C.

### Statistics

GraphPad Prism 5 was used for *t*-test analysis in all figures except in Figure 5 where a two-way ANOVA with Bonferroni was used.
